# The ketogenic diet influences taxonomic and functional composition of the gut microbiota in children with severe epilepsy

**DOI:** 10.1038/s41522-018-0073-2

**Published:** 2019-01-23

**Authors:** Marie Lindefeldt, Alexander Eng, Hamid Darban, Annelie Bjerkner, Cecilia K Zetterström, Tobias Allander, Björn Andersson, Elhanan Borenstein, Maria Dahlin, Stefanie Prast-Nielsen

**Affiliations:** 1 Neuropediatric Department, Astrid Lindgren Children’s Hospital, Karolinska Hospital, Stockholm, Sweden; 2 Department of Genome Sciences, University of Washington, Seattle, WA 98195 USA; 3 Department of Cell and Molecular Biology, Karolinska Institutet, Stockholm, Sweden; 4 Department of Clinical Microbiology, Karolinska University Hospital, Stockholm, Sweden; 5 Pediatric Gastroenterology, Astrid Lindgren Children’s Hospital, Karolinska University Hospital, Stockholm, Sweden; 6 Department of Computer Science and Engineering, University of Washington, Seattle, WA 98195 USA; 7 Blavatnik School of Computer Science, Tel Aviv University, 6997801 Tel Aviv, Israel; 8 Sackler Faculty of Medicine, Tel Aviv University, 6997801 Tel Aviv, Israel; 9 Santa Fe Institute, Santa Fe, NM 87501 USA; 10 Center for Translational Microbiome Research (CTMR), Department of Microbiology, Tumor and Cell Biology, Karolinska Institutet, Stockholm, Sweden

## Abstract

The gut microbiota has been linked to various neurological disorders via the gut–brain axis. Diet influences the composition of the gut microbiota. The ketogenic diet (KD) is a high-fat, adequate-protein, low-carbohydrate diet established for treatment of therapy-resistant epilepsy in children. Its efficacy in reducing seizures has been confirmed, but the mechanisms remain elusive. The diet has also shown positive effects in a wide range of other diseases, including Alzheimer’s, depression, autism, cancer, and type 2 diabetes. We collected fecal samples from 12 children with therapy-resistant epilepsy before starting KD and after 3 months on the diet. Parents did not start KD and served as diet controls. Applying shotgun metagenomic DNA sequencing, both taxonomic and functional profiles were established. Here we report that alpha diversity is not changed significantly during the diet, but differences in both taxonomic and functional composition are detected. Relative abundance of bifidobacteria as well as *E. rectale* and *Dialister* is significantly diminished during the intervention. An increase in relative abundance of *E. coli* is observed on KD. Functional analysis revealed changes in 29 SEED subsystems including the reduction of seven pathways involved in carbohydrate metabolism. Decomposition of these shifts indicates that bifidobacteria and *Escherichia* are important contributors to the observed functional shifts. As relative abundance of health-promoting, fiber-consuming bacteria becomes less abundant during KD, we raise concern about the effects of the diet on the gut microbiota and overall health. Further studies need to investigate whether these changes are necessary for the therapeutic effect of KD.

## Gut–brain axis: The ketogenic diet alters functional gut microbiota

The ketogenic diet changes both the relative abundance of gut microbiota and their metabolic activities. The diet forces a shift from carbohydrates to ketones as a primary energy source and has demonstrated efficacy in reducing epileptic seizures in children. After animal models implicated gut microbiota in this amelioration, Stefanie Prast-Nielsen, of Sweden’s Karolinska Institutet, and her team sequenced microbiotic DNA of fecal samples from 12 children with epilepsy before and after 3 months on a ketogenic diet. Changes included reductions in the numbers of *Bifidobacterium* and an increase in *Escherichia coli*. Carbohydrate metabolism significantly changed after 3 months on the diet. Some reductions raise questions about the diet’s potential impact on gut and overall health. More studies are also needed to discern the mechanistic impact of these changes on seizure activity.

## Introduction

The human gut microbiota has received increasing attention in recent years and numerous studies have demonstrated its role in health and disease. Dysbiosis, disturbances in the gut microbiome, has been linked to neurological disorders, such as autism,^1^ anxiety, and depression^2^ via the “microbiome–gut–brain axis.”^3^ This axis is a bidirectional communication system between the intestinal microbiome and the central nervous system involving neural, endocrine, and immune pathways.^4^ As evidence of this, germ-free mice display deficits in brain development and behavior.^5^

Diet influences the composition of the human gut microbiome.^6^ Based on the anti-seizure effect of fasting, the ketogenic diet (KD), a high-fat, adequate-protein, very low-carbohydrate diet, was developed in the early 1920s^7^ to mirror the key metabolic effects of fasting. A diet very high in fat and low in carbohydrates induces multiple changes in the intermediary metabolism and results in the use of ketones as the main energy substrate. In children, KD is used in the treatment of therapy-resistant epilepsy and in neurometabolic disorders where glucose is not fully available as an energy substrate such as glucose transporter type 1 (GLUT1) deficiency syndrome and pyruvate dehydrogenase deficiency. A seizure reduction of >50% has been found in about half of the treated children.^8,9^

Today, the classic KD is calculated with a ratio between 2:1 and 4:1 of fat to protein and carbohydrates combined. The ratio 4:1 contains 4 parts of fat and 1 part of proteins and carbohydrates together (in g). With a 4:1 ratio, 70–90% of energy intake is derived from fat. Despite a long history of clinical use, the mechanisms underlying the seizure-suppressive action are unclear. Several hypotheses have been proposed including changes in neurotransmitter systems, inhibitory action of polyunsaturated fatty acids, or enhancement of mitochondrial function.^10^ Different aspects of human metabolism may be implicated, but little is known about how KD influences our “second genome,” the microbiome. In the present study, we examine how the fecal microbiome is affected by KD in children with epilepsy.

Here we have analyzed both taxonomic composition and functional profiles, i.e., gene content and pathway abundances in the gut microbiome during KD in children with therapy-resistant epilepsy using shotgun metagenomic sequencing.

## Results

Twelve patients starting KD and 11 healthy parents not starting KD were enrolled. For inclusion criteria of the patients, demographics, and treatment details, see Methods and Table 1. Fecal samples were collected at two time points, before and 3 months after starting KD. At the second time point, the ketogenic ratio was 4:1 in 7 children, 3.5:1 in 2, and 3:1 in 3. The ketone levels of β-hydroxybutyric acid were 0.3 ± 0.2 (mean ± SD), range 0.1–0.8 before diet start and 4.1 ± 1.2 with a range of 1.4–5.6 mmol/l after 3 months. Blood glucose levels decreased from 4.9 ± 0.5 (mean ± SD) to 4.3 ± 0.4 mmol/l during the intervention.

**Table 1:** Patients included in the study (*n* = 12) and efficacy concerning seizures and behavior at 3 months on ketogenic diet

Pat. no.	Age at KD start (years)	Sex (M/F)	Age at seizure start (years)	Type of seizures	Etiology	Concomitant AEDs	Previous AEDs (no.)	Comorbidity	Gastrostomy (Y/N)	Efficacy at 3 months		KD at 3 months	
										Seizures	Behavior	Ratio	Blood ketones
1	10.3	F	0.8	Ton, GTC	Prem, asphyxia, CNS bleeding	OXC	7	ID, CP	Y	Resp	Resp	4.0: 1	3.9
2	15.3	F	0.1	Fw, GTC	PDH deficiency	LTG	1	ID, CP	Y	Resp	Resp	3.0: 1	1.4
3	14.2	F	0.6	Fw, GTC	Unknown	CBZ, LAC	6	ID, ADHD	N	Nonresp	Resp	3.5: 1	4.9
4	2.8	M	0.4	Myocl-aton	Lissencephaly	VPA, VGB, TPM	5	ID, hypotonia	Y	Resp	Resp	4.0: 1	5.6
5	8.2	M	0.8	Epil spasms, Fw	Tuberous sclerosis complex	VPA, TPM, LAC	9	ID	N	Nonresp	Resp	4.0: 1	4.6
6	7.9	F	5.8	Ton, Fw	Cerebral infarction	LAC	4	ASD, ADHD, CP	N	Nonresp	Resp	4.0: 1	3.8
7	2.2	M	0.1	Atyp abs, GTC	Genetic mutation	VGB, CLB	8	ID, ataxi	N	Nonresp	Nonresp	4.0: 1	5.3
8	4.9	F	2.3	Ton	Influenza B encephalitis	TPM, CLB	8	ID	N	Resp	Resp	4.0: 1	5.2
9	3.8	F	3.3	Epil spasms	Unknown	VPA, TPM	5	0	N	Nonresp	Nonresp	4.0: 1	4.3
10	11.8	F	5.5	Atyp Abs, CSWS	Unknown	VPA	6	ID, ADHD	N	Nonresp	Resp	3.0: 1	3.9
11	7.6	F	4.1	GTC, CSWS	Unknown	VPA	3	Learning disability	N	Nonresp	Resp	3.0: 1	4.0
12	3.0	M	1.0	Epil spasms	Cortical dysplasia	VPA, LTG	6	ID, ASD	N	Resp	Resp	3.5: 1	2.7

Blood ketones are given in mmol/L

Sex: *M* male, *F* female

Type of seizures: *Ton* tonic, *GTC* generalized tonic-clonic, *Fw* focal with impaired consciousness, *Myocl-aton* myoclonic-atonic, *Epil spasms* epileptic spasms, *Atyp Abs* atypical absences, *CSWS* continuous spike-wave during sleep

Etiology: *Prem* prematurity, *PDH* pyruvate dehydrogenase deficiency

AEDs: *OXC* oxcarbazepine, *LTG* lamotrigine, *CBZ* carbamazepine, *LAC* lacosamide, *VPA* valproic acid, *VGB* vigabatrin, *TPM* topiramate, *CLB* clobazam

Comorbidity: *ID* intellectual disability, *CP* cerebral palsy, *ADHD* attention-deficit hyperactivity disorder, *ASD* autism spectrum disorder

Gastrostomy: *Y* yes, *N* no

Efficacy: *Resp* responder: >50% seizure reduction on KD treatment at 3 months, *Nonresp* non-responder: <50% seizure reduction on KD treatment at 3 months

Five out of the 12 children were responders concerning seizure frequency with >50% seizure reduction. Three patients, though non-responders concerning seizure frequency, had shorter seizures and less postictal tiredness. Ten children were responders concerning cognition and motor function (see Methods). The two children who did not improve in these aspects were non-responders also concerning seizure frequency and tapered the diet after follow-up at 3 months.

### Sequencing results and evaluation

Average sequencing depth was 2.24 ± 0.59 (mean ± SD) million reads for patients and 2.05 ± 0.75 (mean ± SD) million reads for healthy parents. Raw read length was 2 × 300 bp.

A mock community (ZymoResearch) was processed and sequenced identically to all samples. It was used to test marker-based (MetaPhlAn2 and metagenomic operational taxonomic unit (mOTU)), alignment-based (RTG), and kmer-based (kaiju) approaches for taxonomic profiling. Our mock community sequencing data was best recaptured using marker-based taxonomic profiling (Supplementary Figure 1), which was then used for further analysis. We demonstrated that our sample preparation workflow ensured efficient lysis and DNA extraction from different bacterial taxa including both Gram-positive and Gram-negative organisms. However, no fungi from the mock community were identified using any bioinformatics tool tested. A negative control was included in our sample preparation and sequencing procedure, from which 722 paired sequencing reads were obtained, with 428 reads passing quality filtering. None of the reads from the negative control could be identified as a clade-specific marker in MetaPhlAn2.

Raw sequence analysis output tables of taxonomic (MetaPhlAn2) and functional (SUPERFOCUS) profiles are provided as Supplementary Table 1 and Supplementary Table 2.

### Gut microbial diversity

#### Alpha diversity

**Fig. 1:**
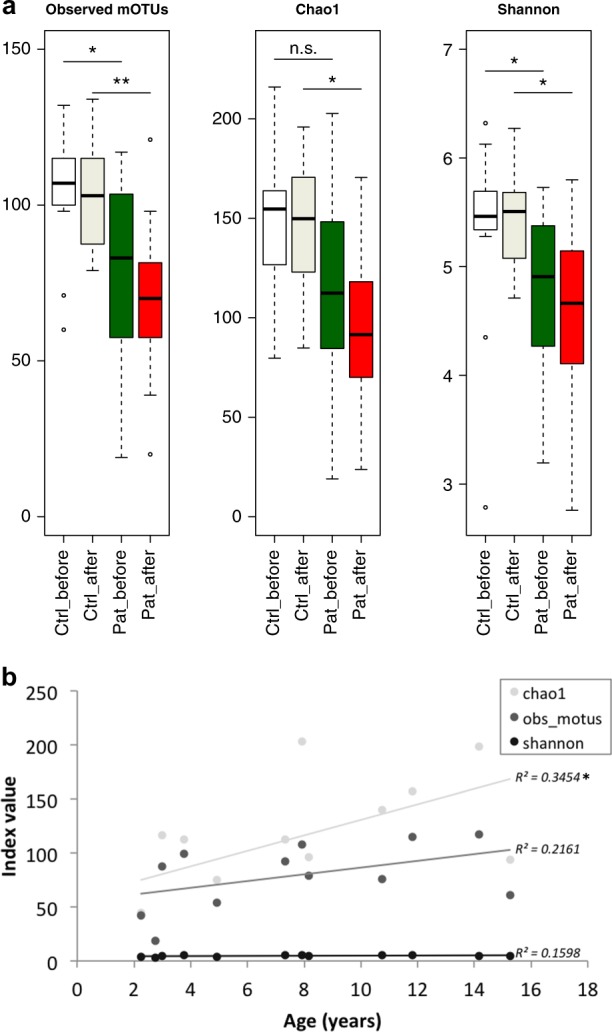
Microbial alpha diversity analysis. From left to right: Total number of observed metagenomic operational taxonomic units (mOTUs), total species richness Chao1, and Shannon’s diversity index of evenness **a**. Data are presented as follows: center line, median; box limits, upper and lower quartiles; whiskers, 1.5× interquartile range; points, outliers. Dunn’s test of multiple comparisons with Benjamini–Hochberg adjustment: *p < 0.05, **p < 0.01. Ctrl control, Pat patient. White, Controls' time point 1; Ivory, Controls' time point 2; Green, Patients' time point 1; Red, Patients' time point 2. Correlation of alpha diversity to age in patients **b** with *R*^2^ values as indicated

Alpha diversity indicates species diversity within a single sample. Observed mOTUs, Chao1, and Shannon diversity were lower in patients than in controls already before treatment, i.e., at time point one, and the difference seemed to increase further after treatment, i.e., at time point two (Fig. 1a). However, the difference in diversity between the two time points was not significant in either patients or controls. Thus introduction of KD had little impact on the patients’ total gut microbial diversity. A slight correlation between alpha diversity values and age of the patients at time point one was detected (Fig. 1b). This was only significant for Chao1 (*p* = 0.044, *r*^2^ = 0.345). Thus a higher alpha diversity in the parents may reflect a more mature gut microbiota.

#### Beta diversity

**Fig. 2:**
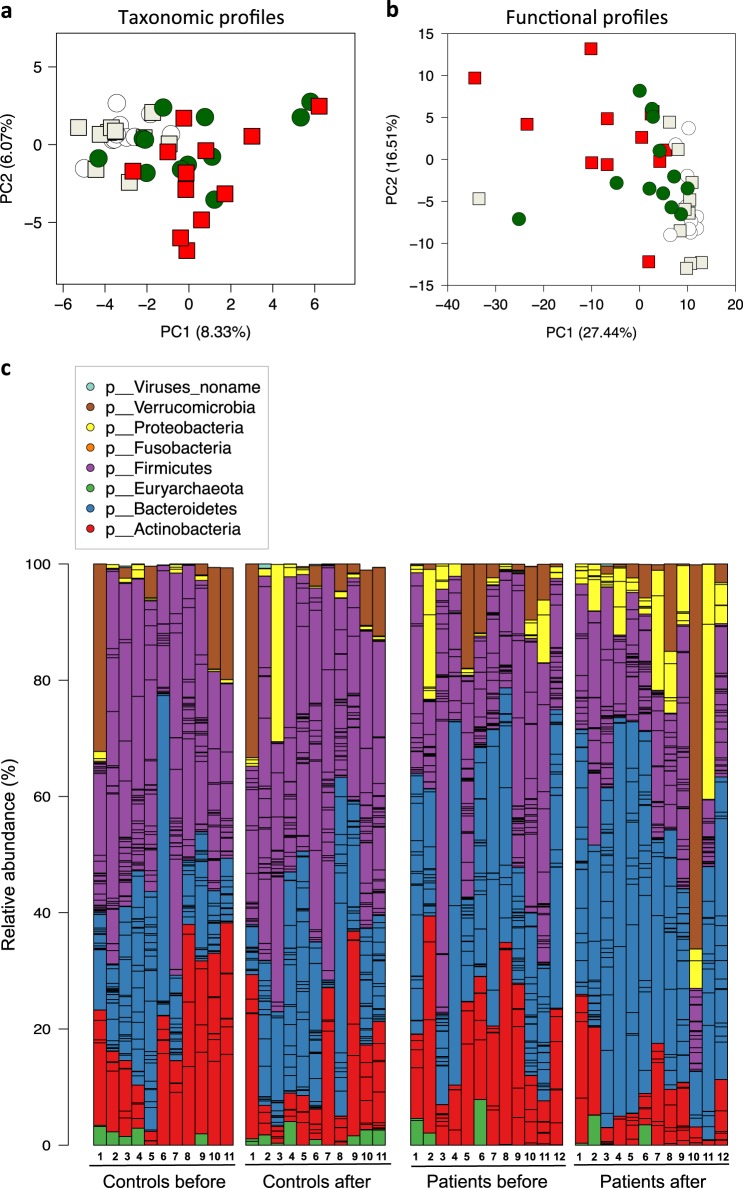
Microbial beta diversity analysis. Principal component analysis (PCA) of **a** taxonomic and **b** functional profiles. Controls before (white circles), Controls after (ivory squares), Patients before (green circles), Patients after (red squares). Taxonomic profiles of individuals at the phylum level **c**

Beta diversity measurements reflect compositional differences between samples. Principal component analysis (PCA) of the taxonomic composition of the gut microbiota revealed clustering of control samples and some clustering of patient samples but patient samples were in general more different from each other than control samples (Fig. 2a). A shift along the *x* axis was detected at time point one in the patients compared to controls, and a shift along the *y* axis was detected at time point two in the patients. This indicates that the gut microbiota in patients was different from that of controls and that introduction of KD has an impact on the composition of the gut microbiota.

PCA of the functional composition of the gut microbiota (Fig. 2b) at SEED subsystem level 3 reproduced clustering of controls and less clustering of patients as seen in Fig. 2a. At time point one, patient samples were shifted along both *x* and *y* axis compared to controls and at time point two this shift was even more pronounced, indicating differences in the functional profile of the gut microbiota between patients and controls and that KD influences the functional composition.

### Analysis of taxonomic changes in the gut microbiota during KD

Dominant phyla in fecal samples from patients and controls at time point one were Firmicutes, Bacteroidetes, and Actinobacteria (Fig. 2c), which are typically found in the human gut.^11^ Here intraindividual changes were detected in both groups. In the control group relative abundance of Actinobacteria, had decreased in several individuals. Other phyla appeared more stable in the parents compared to the patients, where we observed both a decrease in relative abundance of Actinobacteria and Firmicutes and an increase in relative abundance of Bacteroidetes and Proteobacteria.

**Fig. 3:**
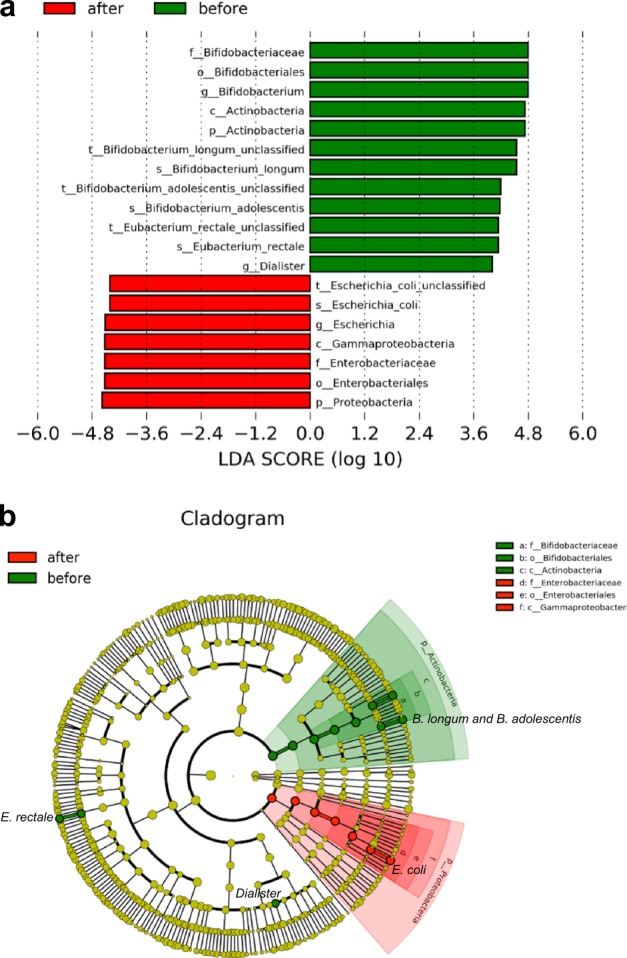
Statistical analysis of taxonomic profiles. Significant changes at all taxonomic levels during dietary intervention (KD) using the linear discriminant analysis (LDA) effect size (LEfSe) method; *p* < 0.05, LDA > 4.0 **a**. Cladogram of significant changes at all taxonomic levels during KD **b**. Green: Patients' time point 1, Red: Patients' time point 2

Next, we applied the linear discriminant analysis (LDA) effect size (LEfSe) method by Segata et al.^12^ for statistical analysis at all taxonomic levels. Nineteen discriminative features at various taxonomic levels were detected (Fig. 3a). At the phylum level, relative abundance of Actinobacteria was significantly diminished and Proteobacteria increased proportionally 3 months after starting KD. The decrease in Actinobacteria relative abundance could mainly be attributed to a decrease of relative abundance of the genus *Bifidobacterium* (Fig. 3b), with a mean relative abundance before KD: 15.8%; 3 months into KD: 3.9%. Within the genus *Bifidobacterium*, two species were significantly decreased: *Bifidobacterium longum* (mean relative abundance before KD: 8.1%; 3 months after starting KD: 2.4%) and *B. adolescentis* (before KD: 3.2%; 3 months into KD: 0.2%). The mean relative abundance of *Eubacterium rectale* and genus *Dialister* also decreased during KD (2.5% before; 0.5% after, and 2.2% before; 0.4% after, respectively). Genus *Escherichia* was more relative abundant 3 months after starting KD (mean: 3.1% before; 8.5% after), which was largely due to an increase of *Escherichia coli*.

Applying the same parameters to the parental dataset, relative abundance of one unclassified strain of *Eubacterium siraeum* was significantly increased from time point one to two. None of the features found significantly changed in the patients were identified here.

### Analysis of functional changes in the gut microbiota during KD

Significant functional changes in subsystem relative abundances and effect size were calculated (Table 2). Twenty-nine subsystems changed significantly due to KD. Relative abundance of 26 pathways was diminished after 3 months on KD and 3 became more relative abundant. The group with most pathways changed was carbohydrate metabolism, showing reduction of fructooligosaccharides (FOS) and raffinose utilization, sucrose utilization, glycogen metabolism, lacto-*N*-biose I and galacto-*N*-biose metabolic pathway; Fermentations: lactate, pentosephosphate pathway; and formaldehyde assimilation: ribulose monophosphate pathway. Analysis of the parental dataset did not detect any discriminative features at either *p* < 0.01 or *p* < 0.05 and LDA > 2.0.

**Table 2:** Significant functional changes at SEED level 3 during KD using the LDA effect size method

SEED subsystem level 1	SEED subsystem level 2	SEED subsystem level 3	Enriched	LDA score (log 10)	*p* Value
Carbohydrates	Disaccharides and oligosaccharides	Fructooligosaccharides (FOS) and raffinose utilization	Before	2.92	0.009
Carbohydrates	Disaccharides and oligosaccharides	Sucrose utilization	Before	2.69	0.001
Carbohydrates	Polysaccharides	Glycogen metabolism	Before	2.88	0.009
Carbohydrates	—	Lacto-*N*-biose I and Galacto-*N*-biose metabolic pathway	Before	2.64	0.004
Carbohydrates	Fermentation	Fermentations: lactate	Before	2.31	0.003
Carbohydrates	Central carbohydrate metabolism	Pentosephosphate pathway	Before	2.14	0.008
Carbohydrates	One-carbon metabolism	Formaldehyde assimilation: ribulose monophosphate pathway	Before	2.07	0.007
Clustering-based subsystems	—	Bacterial cell division	Before	2.53	0.008
Clustering-based subsystems	—	RP bacterial cell division	Before	2.53	0.009
Clustering-based subsystems	Tricarboxylate transporter	CBSS-49338.1.peg.459	Before	2.32	0.008
Clustering-based subsystems	Proteosome related	Cluster-based subsystem grouping hypotheticals—perhaps proteosome related	Before	2.01	0.009
Cofactors, vitamins, prosthetic groups, pigments	Riboflavin, FMN, FAD	Test—Riboflavin	Before	2.61	0.003
Cofactors, vitamins, prosthetic groups, pigments	—	Test—Thiamin	Before	2.40	0.007
Cofactors, vitamins, prosthetic groups, pigments	Coenzyme A	coA-FolK	Before	2.21	0.004
Cell division and cell cycle	—	Two cell division clusters relating to chromosome partitioning	Before	2.78	0.008
Cell division and cell cycle	—	Bacterial cytoskeleton	Before	2.51	0.001
Regulation and cell signaling	Regulation of virulence	Streptococcal Mga regulon	Before	2.03	0.004
Regulation and cell signaling	—	Cell envelope-associated LytR-CpsA-Psr transcriptional attenuators	Before	2.03	0.003
DNA metabolism	DNA repair	DNA repair, bacterial RecBCD pathway	Before	2.81	0.007
DNA metabolism	DNA repair	DNA repair, bacterial	Before	2.54	0.006
Virulence	Resistance to antibiotics and toxic compounds	*Streptococcus pneumoniae* vancomycin tolerance locus	Before	2.19	0.001
Virulence	—	Mycobacterium virulence operon involved in an unknown function with a Jag protein and YidC and YidD	Before	2.16	0.009
Nitrogen metabolism	—	Ammonia assimilation	Before	2.52	0.005
Fatty acids, lipids, and isoprenoids	Fatty acids	Fatty acid biosynthesis FASI	Before	2.43	0.001
Amino acids and derivatives	Lysine, threonine, methionine, and cysteine	Lysine biosynthesis DAP pathway	Before	2.19	0.008
Stress response	Oxidative stress	Redox-dependent regulation of nucleus processes	Before	2.05	0.006
Respiration	Electron-donating reactions	Succinate dehydrogenase	After	2.51	0.009
Iron acquisition and metabolism	—	Hemin transport system	After	2.24	0.003
RNA metabolism	RNA processing and modification	ATP-dependent RNA helicases, bacterial	After	2.11	0.004

SEED subsystem level 3: significantly discriminative features (*p* < 0.01, LDA > 2.0)

Enriched: *before* subsystem enriched before starting KD, *after* subsystem enriched after 3 months on KD

*LDA* linear discriminant analysis

**Fig. 4:**
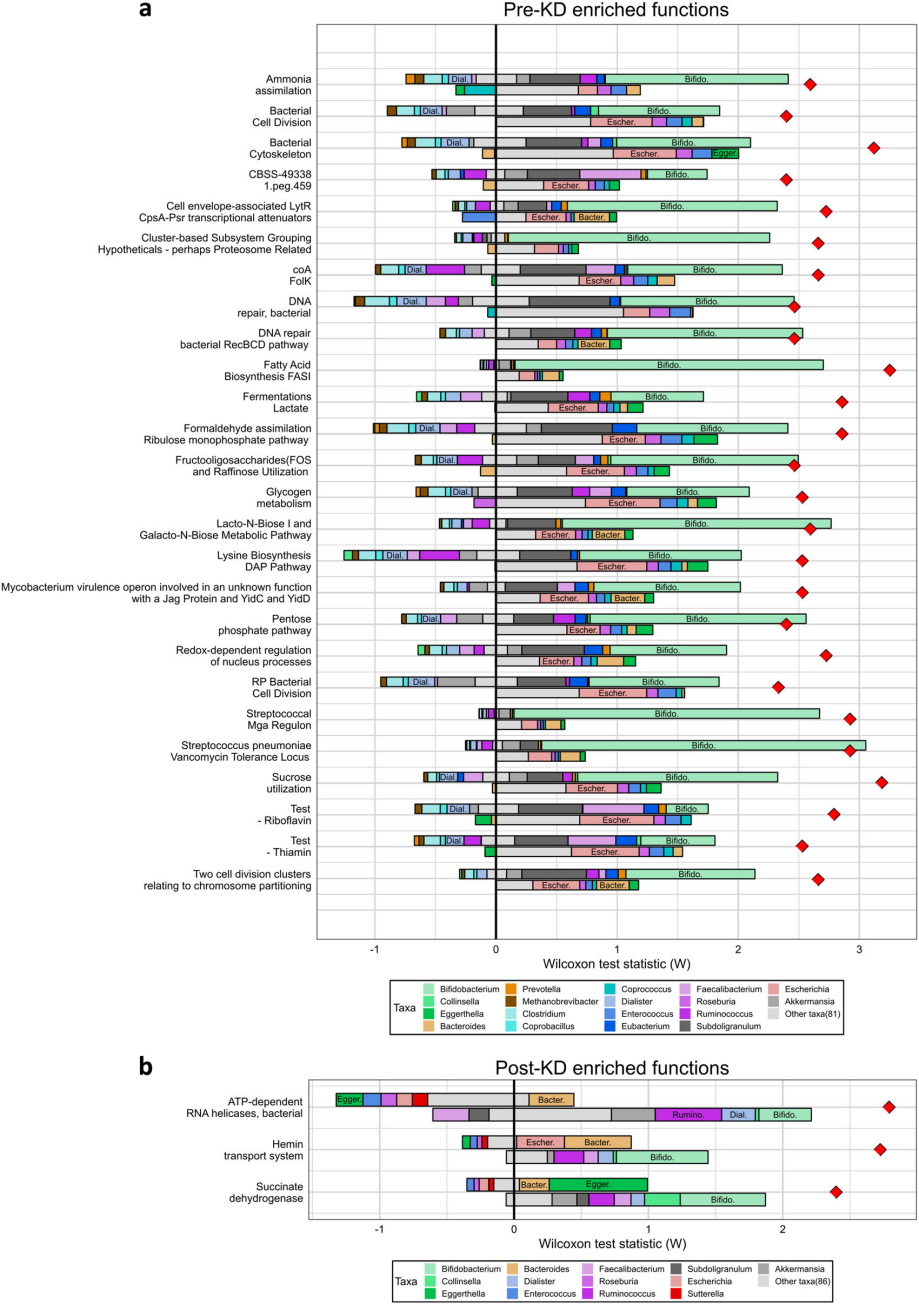
Taxonomic drivers of functional shifts associated with KD. Taxonomic contributions to the shift in each function are shown as bars for functions enriched pre-KD compared to post-KD **a** and functions enriched post-KD compared to pre-KD **b**. Bar length represents the size of the contribution. For each function, the position of the bar indicates the type of contribution. The top (bottom) bars represent contributions from genera with higher (lower) relative abundance in the enrichment group (pre-KD for **a**, post-KD for **b**). Bars to the left (right) of the vertical black line represent contributions to decreased (increased) function abundance in the enrichment group. The red diamonds indicate the observed increase in relative median function abundance in pre-KD samples **a** or post-KD samples **b**. Colors indicate the genus, with genera from the same phylum sharing similar colors: green for Actinobacteria; orange for Bacteroidetes; teal, blue, and purple for Firmicutes; and red for Proteobacteria. Genus labels within bars are provided for genera with notable contributions where bars were wide enough to accommodate labels

Computational decomposition of post-KD functional shifts was used to predict potential taxonomic contributions to observed functional changes. This analysis suggested that the decrease in *Bifidobacterium* relative abundance and increase in *Escherichia* relative abundance both contribute to the decreased relative abundance of multiple pathways, including carbohydrate metabolism pathways (Fig. 4a). In contrast, increases in pathway relative abundance was predicted to be more genus specific. For example, the increase in both *Escherichia* and *Bacteroides* relative abundances was inferred to be a potential driver of the increase in the Hemin transport system pathway, a function that has indeed been previously observed in both *Escherichia*^13–15^ and *Bacteroides*^16–18^ species. Interestingly, this decomposition also inferred that the increase in *Eggerthella* relative abundance might drive the increase in the succinate dehydrogenase subsystem (Fig. 4b). This subsystem contains genes encoding fumarate reductase subunits. According to the KEGG^19^ database annotation of the *Eggerthella lenta* genome, it encodes three sequences related to fumarate reductase subunits.

These links between taxonomic and functional changes in our pilot dataset are based on computational inference and may require validation and confirmation in a larger cohort. However, they are useful to generate intriguing hypotheses concerning the taxonomic drivers of observed functional shifts.

### Analysis of butyrate production potential

**Fig. 5:**
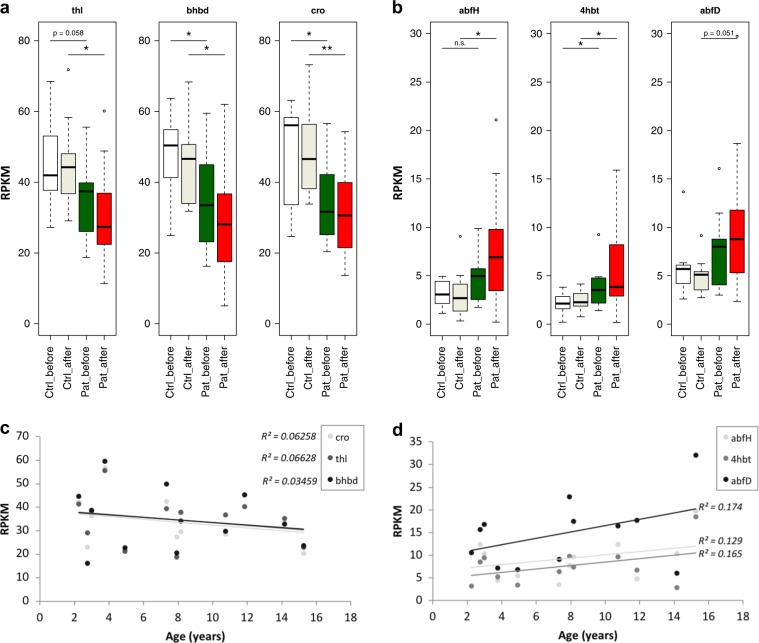
Analysis of butyrate production potential. RPKM (reads per kilo base per million) values for unique genes of the acetyl-CoA pathway **a** and the 4-aminobutyrate pathway **b** for butyrate production. Data are presented as follows: center line, median; box limits, upper and lower quartiles; whiskers, 1.5× interquartile range; points, outliers. Dunn’s test of multiple comparisons with Benjamini–Hochberg adjustment: *p < 0.05, **p < 0.01. Ctrl control, Pat patient. White, Controls time point 1; Ivory, Controls time point 2; Green, Patients time point 1; Red, Patients time point 2. Correlation of RPKM of each gene to age of patients at time point 1 for the acetyl-CoA pathway **c** and the 4-aminobutyrate pathway **d**. None of the correlations were significant (*p* > 0.05). *thl* acetyl-CoA acetyltransferase (thiolase), *bhbd* β-hydroxybutyryl-CoA dehydrogenase, *cro* crotonase, *abfH* 4-hydroxybutyrate dehydrogenase, *4hbt* butyryl-CoA:4-hydroxybutyrate CoA transferase, *abfD* 4-hydroxybutyryl-CoA dehydratase and vinylacetyl-CoA 3,2-isomerase (same gene)

*E. rectale*, a major butyrate-producing species in the human gut, significantly decreased proportionally in patients during KD. To analyze whether this might impact the total butyrate synthesis potential of the whole community, a bacterial butyrate synthesis gene database^20^ was used to identify reads from genes involved in butyrate production in our shotgun metagenomic dataset. RPKM (reads per kilo base per million) values were calculated for all genes of the known microbial butyrate production pathways upstream of *bcd*/*etfAB* (butanoyl-CoA dehydrogenase, E.C.: 1.3.1.109), as there are downstream overlaps between pathways. *gcdB* of the glutarate pathway was excluded owing to recruitment of many false positives.^21^ Four butyrate-producing pathways are currently identified in human gut microbial communities^20^; the acetyl-CoA, the glutarate, the 4-aminobutyrate, and the lysine pathway. KD intervention did not have any overall significant impact on the relative abundance of any of these pathways (Fig. 5 a, b and Fig. Supplementary Figure 2 a, b). Interestingly though, we found that genes from the most abundant butyrate-producing pathway in the healthy human gut microbiome, the acetyl-CoA pathway, were less abundant in our patients compared to controls already before starting KD. Here a very weak but inverse correlation was detected between age of the patients and relative gene abundance indicating that the lower relative abundance of genes of the acetyl-CoA pathway in our patients (Fig. 5c) compared to our controls before KD was not due to the age difference between both groups. Rather, increasing age in our patients might increase the difference to the controls. An opposite trend was shown for the 4-aminobutyrate pathway (Fig. 5d). This pathway was shown to be less relative abundant in the healthy gut microbiome compared to the acetyl-CoA pathway.^20^ In our cohort, genes of this pathway were more frequently identified in patients compared to controls before KD and a possible, yet not significant, further proportional increase was experienced during KD. Relative gene abundance seemed to slightly increase with age of the patients, indicating that this observation cannot be explained by age differences in the patient and control groups either. The lysine pathway did not show any significant differences in relative gene abundances within or between the groups at either time point (Supplementary Figure 2a). In the glutarate pathway, the relative abundance of one out of the six genes (*hgCoAdA*) was significantly decreased in the patients (Supplementary Figure 2b). However, the relative abundance of the concomitant enzymatic subunits *hgCoAdB* and *hgCoAdC* or other genes involved in this pathway was not decreased. It therefore seems unlikely that the glutarate pathway relative abundance is significantly different in any group of samples.

## Discussion

KD has been used successfully as an alternative treatment for therapy-resistant epilepsy since the 1920s. About half of the patients respond to the diet with at least 50% seizure reduction. There have been a number of studies trying to elucidate the mechanisms of action of KD. Evidence for the involvement of the microbiome in seizure susceptibility and KD treatment has recently been presented in animal models. KD demonstrated anti-seizure efficacy in two epilepsy mouse models but not when the mice were raised germ-free.^22^ Seizure susceptibility could be transferred by fecal microbial transplants (FMTs) in rats.^23^ This finding was supported by a single case study where a patient became seizure-free for at least 20 months after an FMT for Crohn’s disease.^24^

We studied taxonomic and functional changes in the fecal microbiome of epilepsy patients treated with KD. Twelve children followed the strict classic KD with a ratio of 3:1 to 4:1. Fecal samples from patients were obtained before and 3 months after starting KD. Eleven healthy parents were sampled at the same time points but did not start KD serving as controls. Whole metagenomic sequencing revealed taxonomic and functional shifts due to KD. The relative abundance of the genus *Bifidobacterium* was significantly decreased in patients after 3 months on KD. The species *B. longum* and *B. adolescentis* were significantly reduced but a similar trend was also observed for other bifidobacteria such as *B. bifidum* and *B. catenulatum* (data not shown).

Bifidobacteria are common to the healthy human gastrointestinal tract. They metabolize complex carbohydrates and possess one of the largest predicted glycobiomes^25^ including genes encoding for a specific hexose fermentation pathway called fructose-6-phosphate shunt or “bifid” shunt.^26^ This pathway is superior in the energy output produced to pathways used by other fermentative gut bacteria and provides a growth advantage for bifidobacteria in the presence of complex carbohydrates. These facts may explain the concomitant proportional decrease of bifidobacteria and genes involved in carbohydrate metabolism during KD.

Duncan et al.^27^ supports our findings of diminished relative abundance of bifidobacteria and *E. rectale* in a low carbohydrate diet as well as our hypothesis that this may result in lower production of acetate and lactate. Acetate and lactate are products of the bifid shunt. Both acids contribute to a decreased pH in the gut, which may prevent pathogen growth. More specifically, Fukuda et al. has shown that bifidobacteria can protect mice from infection by enteropathogenic *E. coli* O157:H7 through production of acetate.^28^ In our study, we show that bifidobacteria relative abundance decreases during KD and *E. coli* relative abundance increases. This may, at least partly, be due to a decreased acetate production by *Bifidobacterium* and/or an increased luminal pH. Agus et al. demonstrated that a Western diet rich in both high fat and high sugar increased the relative abundance of Proteobacteria in mice that correlated with intestinal inflammation and increased susceptibility to adherent-invasive *E. coli* infection.^29^ *E. coli* is a versatile species comprising both commensal and pathogenic strains and can consume carbohydrates from a variety of sources through cross-feeding, including dietary fiber but also shed epithelial cells, and mucosal polysaccharides degraded by intestinal anaerobes.^30^ *E. coli* is associated with a variety of chronic intestinal diseases, including inflammatory bowel disease (IBD).^31^ Therefore, the expansion of *E. coli* during KD might be of concern for general gut health in our patients.

Acetate can be consumed by butyrate-producing gut bacteria. Riviére et al.^32^ demonstrated mutual cross-feeding between *B. longum* and *E. rectale*. This may contribute to the concomitant decrease of *E. rectale* relative abundance in our dataset. The reduced consumption of certain carbohydrates (resistant starch or wheat bran) may also have had a direct effect on the proportional decrease of *E. rectale*.^33,34^ A consequence of the depletion of *Bifidobacterium* and *E. rectale* may be a decreased production of short chain fatty acids (SCFA), specifically acetate in the bifid shunt and butyrate by *E. rectale*. SCFA are important for overall health and low fecal amounts have been associated with diseases such as IBD^35^ and advanced colorectal adenoma.^36^ Both acetate and butyrate have been shown to have anti-inflammatory activity.^37–42^ In addition, butyrate has a direct effect on gut health, being an essential energy source for colonocytes and increasing the intestinal barrier function.^43,44^ In our dataset, the relative abundance of *E. rectale* was rather low in the patients before starting the diet but further decreased during KD. However, the butyrate-producing community is a taxonomically diverse functional group.^20^

Based on our results, we hypothesize that, before starting KD, our epilepsy patients may have a different butyrate production profile than our controls, salvaging relatively more butyrate from the 4-aminobutyrate pathway and less from the acetyl-CoA pathway. This difference may be augmented during KD. Whether epilepsy patients have lower butyrate production in general is not possible to conclude from our dataset. A larger cohort in whom the actual metabolite butyrate is measured will provide more insight into this intriguing finding.

Other studies have recently investigated purely taxonomic changes upon KD treatment of children with GLUT1 deficiency syndrome or epilepsy by either real-time qPCR of selected microbial taxa^45^ or 16S rRNA gene sequencing^46,47^ with inconsistent results. While Tagliabue et al. detect a significant increase in *Desulfovibrio* spp. during KD in an Italian population, their conclusion concurs with ours—a concern about detrimental effects of KD on gut health. They identify a potential need for recommendations on probiotic or prebiotic supplementation. Interestingly, probiotic treatment lead to a >50% reduction in the number of seizures in 28.9% of epilepsy patients in a recent study.^48^ In agreement with Zhang et al., we detect a decrease in alpha diversity and relative abundance of Actinobacteria due to KD but they do not find a proportional increase in *E. coli* in their Chinese cohort.

In our cohort, 5 patients (41.7%) responded with >50% decrease in the number of seizures. However, in 10 children (83.3%) improved cognition and motor functions were observed. Interestingly, Ma et al. showed in mice that KD enhanced neurovascular functions including cerebral blood flow, which may influence cognitive capability.^49^ So far, it is unclear whether this can be observed in humans.

A strength of the present study is the investigation of both taxonomic and functional changes in the gut microbiota during KD. However, we are aware of some weaknesses of our study. Owing to the small size of this pilot cohort (12 patients and 11 controls), we are continuing to recruit patients for a larger study, in which we aim to stratify responders from the non-responders to identify differences in their gut microbiota. Another weakness is the heterogeneity of this epilepsy cohort in which different etiologies were included, and in one third of patients, despite thorough investigations including genetics, a specific etiology could not be obtained. Also the study lacks age-matched controls. Enrollment of healthy children proved difficult, but parents of our patients, sharing the same household, agreed to participate. Even though none of the parents started KD for themselves, they may have changed their dietary habits to some extent, i.e., eating less sugar and more fat. This might explain the decrease in Actinobacteria relative abundance (Fig. 2c), which, however, was not statistically significant. Apart from that, the microbiome of the parents was more stable comparing time point one and two and none of the taxa or pathways found significantly changed owing to KD in patients were found in parents. Importantly, as we aimed to study the effect of KD on the gut microbiota we compare communities and function before versus 3 months after starting KD within the patient group.

Here we show that the fecal microbiome of children with epilepsy changes during KD. The compositional changes observed might not be favorable for gut or overall health based on the current understanding of the composition and role of a healthy gut microbiota. Taxa believed to be health promoting (bifidobacteria and *E. rectale*) decrease in relative abundance and with them their health-promoting metabolites. One hypothesis for the mechanism of action of KD is the restriction of glucose production from dietary carbohydrates and production of ketone bodies from fat as alternative energy sources. While simple dietary sugars highly abundant in a modern Western diet are readily absorbed in the small intestine and contribute to elevated blood glucose levels, complex, so-called non-digestible carbohydrates (NDCs) cannot be hydrolyzed by the human enzyme repertoire and reach the large intestine. Several studies have shown the important role of dietary fibers/NDCs as energy sources for gut microbes in order to maintain gut health.^50–52^ Thus supplementing patients on KD with such fibers might seem advisable. The prebiotics inulin, lactulose, FOS, and galacto-oligosaccharides have been investigated in several human trials, and studies suggest that these carbohydrates preferentially increase bifidobacteria and decrease *E. coli* and enterococci.^53^ This might prevent undesired changes in the gut microbiota in our patients. However, we first need to understand the role that changes in the gut microbiota during KD play in its therapeutic effect. Olson et al.^22^ elegantly showed that a gut microbiota was necessary for the anti-seizure effect of KD in mice. Here, *Akkermansia* and *Parabacteroides* mediated this effect involving changes in systemic gammaglutamylated amino acids and elevated hippocampal GABA/glutamate levels. Identifying which microbial taxa and functions may be correlated to a positive effect of the diet in patients could lead to the development of probiotic supplements for non-responders to increase their chance of response to the intervention. Ideally, KD could even be replaced by the right combination of prebiotic and probiotic supplements or FMTs in the future. A larger cohort is needed to further investigate this. Additionally, while our cohort suffered from therapy-resistant epilepsy, KD has also been shown to be effective in many other diseases, such as cancer,^54–56^ multiple sclerosis,^57^ Alzheimer’s and Parkinson’s,^58^ depression, autism, and type 2 diabetes.^59^ Thus our results may have implications for a wide range of serious health concerns.

## Methods

### Patient cohort

The present study was performed at the Neuropediatric Department, Astrid Lindgren Children’s Hospital, Karolinska Hospital. The patients were enrolled consecutively as they attended the Neuropediatric Outpatient Clinic and a decision was made to start KD. The inclusion criteria were: age 2–17 years; therapy-resistant inoperable epilepsy or a diagnosis of a neurometabolic disorder in which KD is recommended, no medical contraindications to a trial with KD, and consent for fecal sampling. Exclusion criteria were antibiotics taken within 3 months before starting KD.

The cohort included 12 children starting KD. Eleven suffered from therapy-resistant epilepsy and one had a neurometabolic disorder, pyruvate dehydrogenase deficiency. The latter patient also had epilepsy but seizures were infrequent. For demographics, see Table 1. Four were males and eight females. Their age at diet start were 7.7 ± 4.5 (mean ± SD) years. The mean age at seizure start was 2.1 ± 2.1 (mean ± SD) years, (range 0.1–5.8). The types of seizures were classified according to the revised terminology of the International League Against Epilepsy classifications.^60^ The majority of children had more than one seizure type. Etiology could be determined in eight cases. In four patients, genetic mutations had been verified. The cohort had previously been on treatment with a mean of 5.7 ± 2.3 (mean ± SD) anti-epileptic drugs (AEDs), range 1–9. At the time of KD start, the children were all on daily AED treatment. They were treated with mean 1.8 ± 0.8 (±SD), range 1–3 AEDs at diet start. At 3 months on KD, when follow-up of efficacy and second sampling of fecal microbiota was made, all had the same AEDs and dosing as before diet start except for two patients. One had tapered lacosamid soon after diet start (a non-responder) and the other had a slight increase in clobazam dose (a non-responder). Eleven children had been investigated with neuropsychological formal testing methods concerning intellectual ability. Nine patients were diagnosed with intellectual disability (ID) ranging from mild to severe. One child was found to have learning disability and attention problems but no diagnosis was made, one child was late in development and not yet tested, and one child was clinically not found to have any deficits. Other comorbidities as autism spectrum disorder, attention- deficit hyperactivity disorder, and cerebral palsy were also common. Nine patients were fed orally and three patients had a gastrostomy.

Parents of the children included in the cohort acted as controls (one parent per child). The controls had a normal dietary intake with a western diet and no one had any specific diets. They did not make any substantial changes in their diet during the study period.

### Study design

Seizure frequency was determined from seizure calendars in which parents and other caregivers made daily notes of the number and type(s) of seizures. They made notes during the month before KD and during KD. Calculations of these notes were used to define seizure response. Mean seizure frequency the month before starting KD was compared with mean seizure frequency the month before follow-up. Children with <50% seizure reduction were classified as non-responders and those with >50% seizure reduction as responders. Evaluation of cognitive and motor function was based on observations made by the parents and caregivers. They answered a questionnaire before diet start and at 3-month follow-up concerning the level of their child’s alertness, social interest and interaction, verbal responsiveness, ability to communicate, and motor function. Cognitive and motor function was considered improved if clear positive changes were experienced.

We followed a standardized protocol for the classic KD, which is a slightly modified version of the protocol of the Johns Hopkins Hospital.^61^ A dietitian specially trained to carry out KD treatment calculated total calorie level per day and composition of meals and supplements for each individual. These calculations were based on a 2-day diary kept by the parents before admission in which they recorded all food consumed by the child as well as discussions with the parents on the child’s food preferences. We did not use fasting or calorie restriction. A minimum of 1 g/kg body weight per day of protein was used. The children were supplemented with multivitamins and minerals, including potassium, calcium, magnesium, zinc, selenium, and carnitine. Potassium citrate was used to reduce the risk of kidney stones.

KD was started on a ratio of 2:1. This ratio was increased weekly in half steps, i.e., after 1 week of 2:1, it was increased to 2.5:1. Usually an optimal ratio for the individual was reached in 3–6 weeks. This ratio was kept unchanged until 3 months after start when KD was evaluated concerning efficacy and the second fecal sample was taken.

To initiate the treatment, the child was hospitalized for 4 days. Before starting KD, a venous blood sample was obtained in the first morning before breakfast for analyses of glucose and the ketone β-hydroxybutyric acid. Fecal samples were obtained by a swab from the diaper or toilet paper. During the stay, KD was introduced and controlled by clinical examinations and daily blood levels of glucose, β-OHB, and acid–base balance. Keto diet school to teach parents, relatives, and caregivers on various aspects of the diet was carried out. After discharge, blood was sampled to monitor blood ketones, glucose, and acid–base balance at every increase in ratio and at 3 months on KD.

### Ethics

This study was approved by the Ethics Committee of the Karolinska Hospital (“Regionala etikprövningsnämden i Stockholm”, Dnr 2014/1177-32). Written informed consent was obtained from the legal guardians of the children and, when possible, the children themselves. This research was conducted in accordance with all relevant guidelines and procedures.

### Sample collection, transport, and storage

Fecal sampling was done by the parents using a sterile FLOQSwab™ (Copan). The first sample was taken during the day before starting KD or in the morning of the day of starting. The second sampling at 3 months on diet was collected at home and the swabs were kept in a refrigerator for a few hours until transported to the hospital on ice where all samples were stored at −70 °C. The parents acting as controls delivered their fecal samplings the same day as the child using the same sampling procedure, transport, and storage.

### Sample extraction, processing, sequencing

#### DNA extraction

Total nucleic acids were extracted from frozen fecal samples using the PowerMicrobiome™ RNA Isolation Kit (Qiagen) according to the manufacturer’s instructions and eluted in 108 µl elution buffer. An aliquot of 50 µl was incubated with 10 ng PureLink RNase A (Invitrogen) at 37 °C for 30 min. Subsequently, 5.1 µl sodium acetate (3 M, pH 6.8) and 102 µl isopropanol were added. After 10-min incubation on ice, the DNA was pelleted and washed in ethanol (70% v/v), centrifuged, and dried at room temperature. Finally, purified DNA was re-dissolved in 50 µl RNase-free water and stored at −80 °C.

#### DNA library preparation and sequencing

DNA was quantified using the Qubit dsDNA HS Assay Kit (Thermo Fisher Scientific). 250 ng DNA was sheared in the Covaris® S2 instrument (Covaris, Inc.) to an insert size of approximately 650 bp. Fifty nanograms of sheared DNA was used for preparation of sequencing libraries with the ThruPLEX® DNA-seq Kit (Rubicon Genomics) according to the manufacturer’s instructions using provided primers with ten cycles for amplification. Primer sequences were as follows: 5’: AATGATACGGCGACCACCGAGATCTACACNNNNNNNNACACTCTTTCCCTACACGACGCTCTTCCGATCT with NNNNNNNN being a TruSeq HT i5 index and 3’: GTTCGTCTTCTGCCGTATGCTCTANNNNNNNNCACTGACCTCAAGTCTGCACACGAGAAGGCTAGA with NNNNNNNN being a TruSeq HT i7 index.

PCR products were purified on the MBS Magnatrix 1200 automated workstation (NorDiag) with Dynabeads® MyOne carboxylic acid beads (Thermo Fisher Scientific). Purity of the samples and insert size distribution were inspected using the High Sensitivity DNA Kit on the Agilent 2100 Bioanalyzer instrument (Agilent Technologies). DNA libraries were quantified using the Qubit dsDNA HS Assay Kit (Thermo Fisher Scientific) and equimolar concentrations of 12 samples were pooled and further purified using Agencourt AMPure XP (Beckman Coulter, Inc.). Library pools with a final concentration of 10 nM DNA were submitted to one flow cell per pool and sequenced using the MiSeq V3 chemistry (Illumina).

### Bioinformatics analyses

BBDuk^62,63^ was used to quality filter and trim raw reads with the following parameters: ktrim = r k = 23 mink = 6 hdist = 1 qtrim = rl trimq = 20 minlength = 70 tpe tbo. The quality of the sequences before and after was visually inspected using MultiQC.^64^ Human reads (“contamination”) were removed by alignment to the masked Human Genome version 19 (hg19) in BBMap^63^ using parameters as follows: minid = 0.95, qtrim = rl, trimq = 10, untrim. Patients and controls had <4% human reads, the mock community (ZymoResearch) contained 0.0001% human reads, and the negative control 8.4% (36/428 read pairs total).

We compared marker-based (MetaPhlAn2^65^ and mOTU^66^), alignment-based (RTG^67^), and kmer-based (kaiju^68^) approaches for taxonomic profiling using a mock community from ZymoResearch. MetaPhlAn2 and mOTU were run with default settings. In kaiju, we used the proGenomes reference database for classification in Greedy run mode with -a greedy -e 3 allowing for maximum three mismatches. RTG was run using the default composition-meta-pipeline.

For functional profiling, we applied SUPERFOCUS (SF)^69^ to align our reads against a reduced SEED database^70^ using DIAMOND^71^ and default values.

Alpha diversity was calculated on taxonomic mOTU counts using Qiime v1.9.1 and plotted in R (v.3.4.3 GUI 1.70 El Capitan build). Dunn’s test of multiple comparisons with Benjamini–Hochberg adjustment was performed for significance testing. For further taxonomic analyses, we used MetaPhlAn2 relative abundances. To analyze beta diversity of both taxonomic and functional profiles, we performed PCA in R. Barplots at the phylum level were produced using RColorBrewer and rafalib libraries. LEfSe was used for statistical analysis at all the taxa level and SEED subsystem level 3. Pearson’s product–moment correlation coefficient was calculated in R to test for association between age of the patients and alpha diversities or RPKM values of genes of butyrate production pathways. Functional shift decomposition was performed using FishTaco,^72^ a permutation-based method to estimate taxonomic contributions to functional shifts using Shapley value analysis. Shifts were decomposed at the genus level with genomic content inferred from the MetaPhlAn2 taxonomic profiles and SF functional profiles.

### Butyrate production potential

A bacterial butyrate synthesis gene database encompassing 19,284 known genes was downloaded at http://193.175.244.101/Butyrate/.^20^ Metagenomic reads were aligned using bowtie2 with the --very-sensitive-local setting after removing possible false positives as described by Vital et al. Mapped reads were retrieved using SAMtools (v.1.5, SAMtools view -S -F 4^73^). RPKM values were calculated by dividing the number of reads mapped to each gene by the mean kilo base length of that gene as well as the total number of reads in that sample times 1,000,000. The results were plotted in R and Dunn’s test of multiple comparisons with Benjamini–Hochberg adjustment was applied.

## Supplementary information

Supplementary Information

Supplementary Table 1

Supplementary Table 2

## Data Availability

Raw sequencing data have been submitted to the European Nucleotide Archive under study accession number PRJEB28847.

## Supplementary information

**Supplementary information** accompanies the paper on the *npj Biofilms and Microbiomes* website (10.1038/s41522-018-0073-2).
