# Supplementary material for: The ketogenic diet influences taxonomic and functional composition of the gut microbiota in children with severe epilepsy
Source: NPJ Biofilms Microbiomes. 2019 Jan 23;5:5. doi: 10.1038/s41522-018-0073-2 (PMC6344533; doi:10.1038/s41522-018-0073-2)
Supplement: Supplementary file 1 — Supplementary Information [file 41522_2018_73_MOESM1_ESM.pdf]

Supplementary Information:
The Supplementary Information contains two figures and two tables:

Supplementary Figure 1: Taxonomic analysis of a mock community
Supplementary Figure 2: Analysis of butyrate production potential

Supplementary Table 1:
Supplementary_Table_S1.xlsx - Metaphlan2 output table
Supplementary Table 2:
Supplementary_Table_S2.xlsx - SUPERFOCUS output table

Supplementary Figures

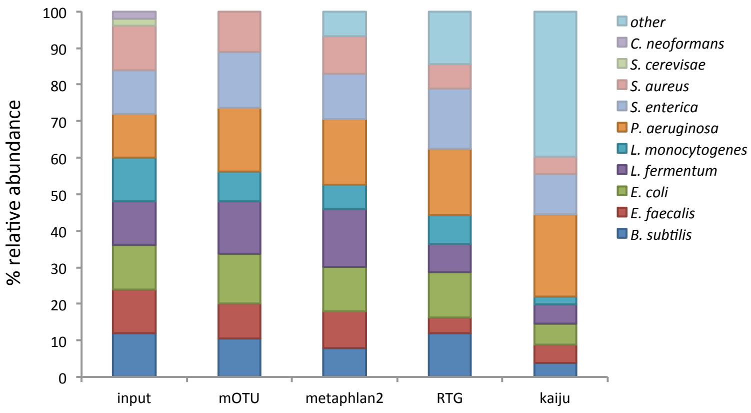

Supplementary Figure 1. Taxonomic analysis of a mock community. A defined microbial community ('input' on x-axis, ZymoResearch) was processed in parallel with fecal samples, thus DNA extraction, library preparation and sequencing was performed identically. The quality-filtered dataset was then used to benchmark our bioinformatics analysis pipeline testing mOTU, metaphlan2, RTG and kaiju. Relative abundance of the expected and other species is presented on the y-axis.

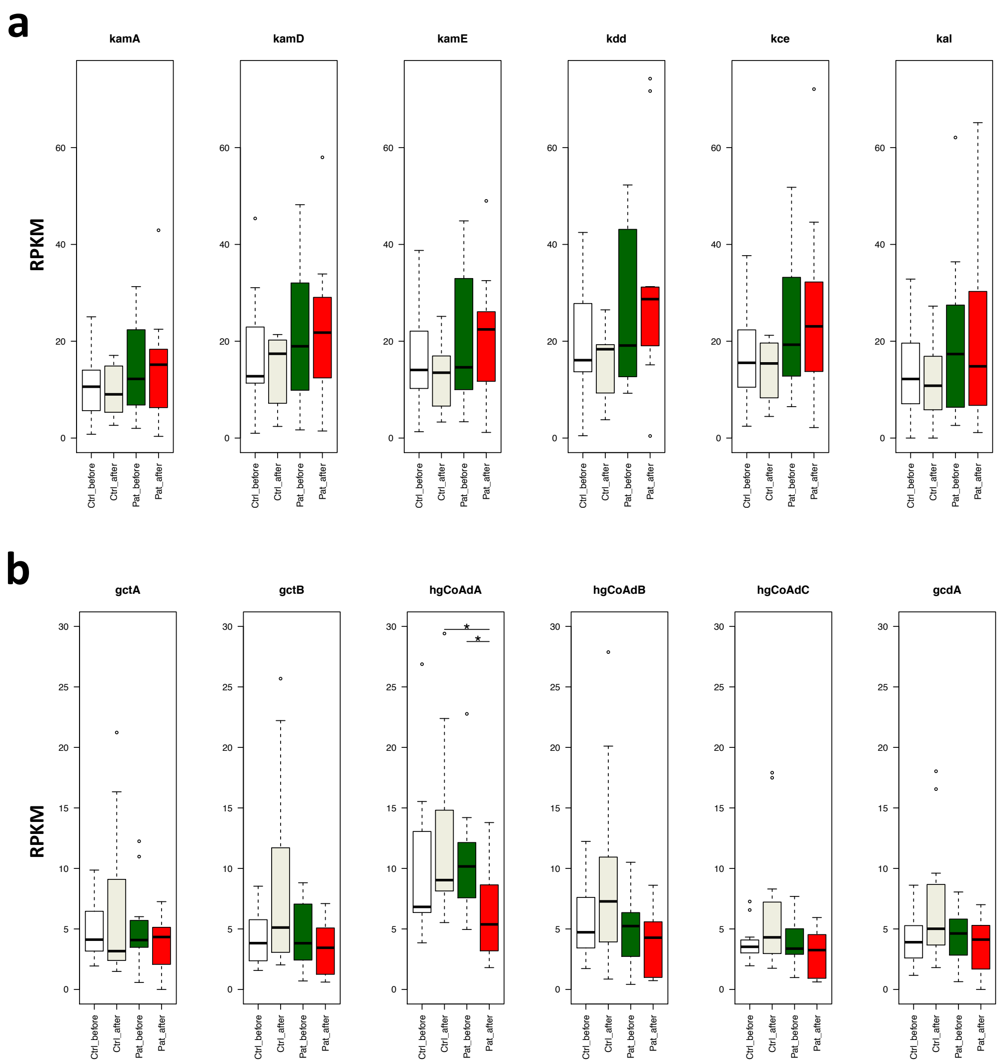

Supplementary Figure 2. Analysis of butyrate production potential. RPKM (reads per kilo base per million) values for unique genes of the lysine pathway (a) and the glutarate pathway (b) for butyrate production. Data are presented as follows: center line, median; box limits, upper and lower quartiles; whiskers, 1.5x interquartile range; points, outliers. Dunn's test of multiple comparisons with Benjamini-Hochberg adjustment: *$p < 0.05$. Ctrl, control; Pat, patient. White, Controls time point 1; Ivory, Controls time point 2; Green, Patients time point 1; Red, Patients time point 2. *kamA*, lysine-2,3-aminomutase; *kamD*, β-lysine-5,6-aminomutase (α subunit); *kamE*, β-lysine-5,6-aminomutase (β subunit); *kdd*, 3,5-diaminohexanoate dehydrogenase; *kce*, 3-keto-5-aminohexanoate cleavage enzyme; *kal*, 3-aminobutyryl-CoA ammonia-lyase; *gctA*, glutaconate-CoA transferase (α subunit); *gctB*, glutaconate-CoA transferase (β subunit); *hgCoAd*, 2-hydroxyglutaryl-CoA dehydratase (α, β, and γ subunit); *gcdA*, glutaconyl-CoA decarboxylase (α subunit).

Supplementary Tables

Supplementary Table 1 and Supplementary Table 2 are provided separately.
